# Conventional Scalpel and Diode Laser Approach for the Management of Traumatic Fibroma

**DOI:** 10.7759/cureus.47810

**Published:** 2023-10-27

**Authors:** Bhashit Diwan, Unnati Shirbhate, Pavan Bajaj, Amit Reche, Abhishek Pahade

**Affiliations:** 1 Dentistry, Sharad Pawar Dental College, Datta Meghe Institute of Higher Education and Research, Wardha, IND; 2 Periodontics, Sharad Pawar Dental College, Datta Meghe Institute of Higher Education and Research, Wardha, IND; 3 Public Health Dentistry, Sharad Pawar Dental College, Datta Meghe Institute of Higher Education and Research, Wardha, IND

**Keywords:** diode laser, aesthetics, management, scalpel, fibroma, surgical excision, traumatic fibroma

## Abstract

An oral fibroma is a benign scar-like reaction frequently resulting from chronic mouth irritation. It is also called an oral polyp, fibrous nodule, localised intraoral fibrous hyperplasia, and traumatic fibroma. Chronic irritation from things like biting one's lips or cheek, orthodontic treatments, rubbing against a hard tooth, or wearing dentures or other dental prostheses is frequently the cause. It is often the same colour as the surrounding mouth lining, but occasionally, it can be paler or appear darker if it has bled. Trauma can cause the surface to become rough and scaly or ulcerated. It is primarily dome-shaped and similar to a pedunculated polyp. A traumatic fibroma most frequently occurs on the inside of the cheek. The inside of the lower lip, the gingiva, and the sides of the tongue are other frequent locations. The given case series reported traumatic fibroma associated with anterior teeth and buccal mucosa treated with a conventional scalpel and diode laser techniques, respectively. Diagnosing and treating the aetiology and educating the patient about the same is essential in fibroma cases. The clinical features of both claims and mere aetiology confirmed the diagnosis. The sole option available when therapy is needed is a surgical fibroma excision. Surgical excision is the most popular method for treating oral or traumatic fibromas. Two ways are available for the surgical removal of an oral fibroma: with a scalpel or using a diode laser. Both case presentations demonstrate that surgical excision with a scalpel and diode laser was discovered to be a simple, efficient, and affordable method for treating traumatic fibroma in this report, which presents two traumatic fibromas with different locations with varying aetiology. Traumatic fibromas reported here were in the aesthetic zones, which need to be treated as they can cause traumatic occlusion and difficulty chewing and speech. The patients reviewed for the next three months revealed complete satisfactory healing and no recurrence in both cases.

## Introduction

Traumatic fibroma is the most common benign lesion of the oral mucosa. In 1967, Barker and Lucas established criteria for identifying true fibromas. It is also known as a traumatic fibroma, focal intraoral fibrous hyperplasia, fibrous nodule or oral polyp, or irritational fibroma. They mainly arise from the gingival connective tissue or periodontal ligament [1]. The growth has a smooth surface, normal-coloured mucosa, a sessile or pedunculated base, and a firm consistency. Due to decreased vascularity, the lesion appears as a round or oval, sessile, broad-based swelling that is painless and has a lighter colour than the surrounding tissue. A smooth-surfaced, hard, asymptomatic nodule with a pink or flesh-coloured tint is the outward manifestation of a clinically traumatic fibroma. From a histopathological perspective, it manifests as a nodule of fibrous connective tissue containing collagen [2]. Fibroma, myxoma, lipoma, and pleomorphic adenoma are differential diagnoses of traumatic fibroma. Complete excision of the lesion is one of the treatment modalities other than excision of the lesion; cryosurgery or intralesional injection of corticosteroids is a different treatment modality that is also performed. The treatment plan modalities require proper blood investigation. There is no recurrence rate after postoperative treatment [3]. Traumatic fibroma involves the buccal cavity; the most common is the tongue. The lesion can be reactive and neoplastic. The aetiological variables that contribute significantly to the recurrence of traumatic fibroma include lip biting, occlusal trauma, improperly aligned teeth, sharp or uneven tooth margins, fractured or faulty restorations, and dental calculus. The lesion has more middle-aged predilection. The advantage of this lesion is that no postoperative scar marks are visible after proper treatment modalities. Oral fibromas grow over weeks or months to reach their maximum size, typically 1 cm in diameter, but vary from case to case. A diagnosis may be suspected if a patient presents with typical symptoms of oral fibroma during a medical examination. To confirm the diagnosis and rule out other conditions, a biopsy may be necessary to remove the lesion. The histological analysis typically reveals dense fibrous tissue with few cells, and the overlying epithelium may appear ulcerated, thin, or thick [4]. This case presentation involved the clinical characteristics of traumatic fibroma and its management with initial therapy, surgical therapy, and removing the aetiology, such as habitual or local factors.

## Case presentation

This report involves two cases of traumatic fibroma associated with the maxillary anterior region and buccal mucosa, which were treated with surgical intervention and reported with aesthetic concern and functionality issues during speech and mastication.

Case 1

**Figure 1:**
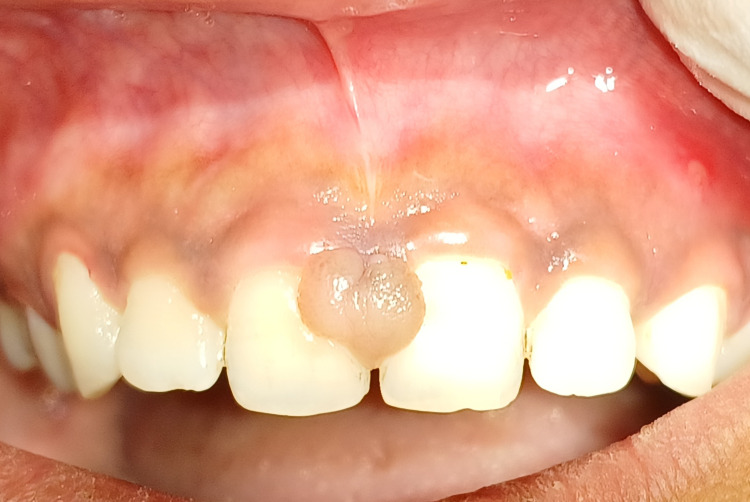
Pre-operative view showing traumatic fibroma associated with anterior teeth.

A 22-year-old female patient reported to the department of periodontics with the chief complaint of gingival swelling in the upper anterior region for two months, approximately 6 mm to 8 mm. Initially, it was small but progressed later. No significant medical history or dental history was present. Considering a history of calculus deposits and traumatic occlusion, the diagnosis of traumatic gingival fibroma was obtained. The lesion was present on the anterior maxillary teeth between the two maxillary central incisors, which was a painless, round, and oval swelling with a broad base and darker colour than the surrounding tissue, as shown in Figure 1.

**Figure 2:**
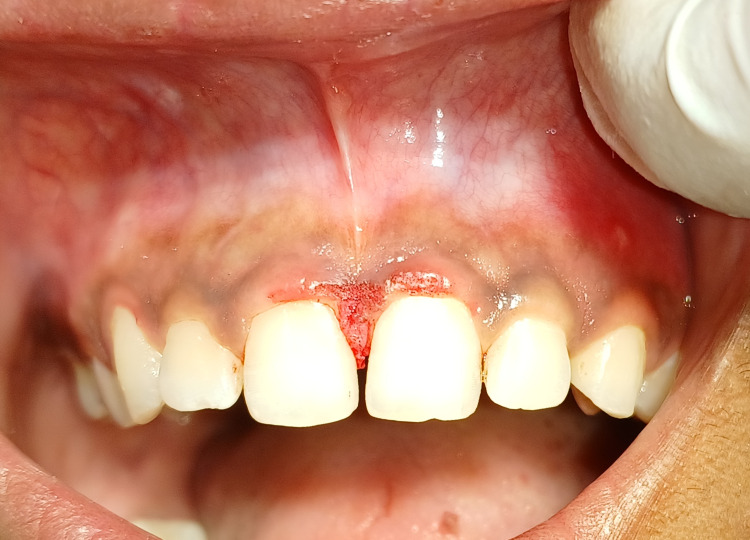
Surgical excision of traumatic fibroma was done by using a scalpel.

Differential diagnoses included fibroma, lipoma, mucocele, and neurofibroma. A provisional diagnosis of traumatic fibroma was obtained based on clinical evidence and patient history. The patient was advised for the surgical removal of the lesion. Routine haematological tests, such as haemoglobin, bleeding and clotting time, and random blood sugar level, revealed test results within normal ranges. Scaling and oral hygiene maintenance counselling were performed before surgical intervention. Informed written consent was obtained. Under all aseptic conditions, precautions, and local anaesthesia, the surgical excision was done using a scalpel, as appreciated in Figure 2. Haemostasis was achieved.

**Figure 3:**
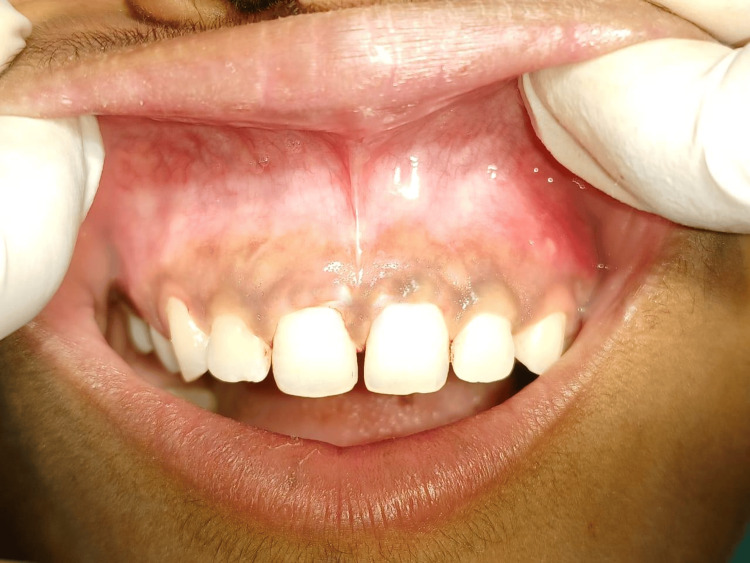
Follow-up examination after seven days revealed complete satisfactory wound healing of the lesion.

An excised lesion specimen was sent for histopathological examination, confirming the diagnosis. Postoperative instructions were given to the patient. The patient was recalled for follow-up evaluation after seven days and three months post-operatively. After seven days, the patient was re-evaluated, which revealed complete recovery and satisfactory healing of the traumatic fibroma lesion, as shown in Figure 3. A follow-up examination after three months revealed no recurrence and good healing.

Case 2

**Figure 4:**
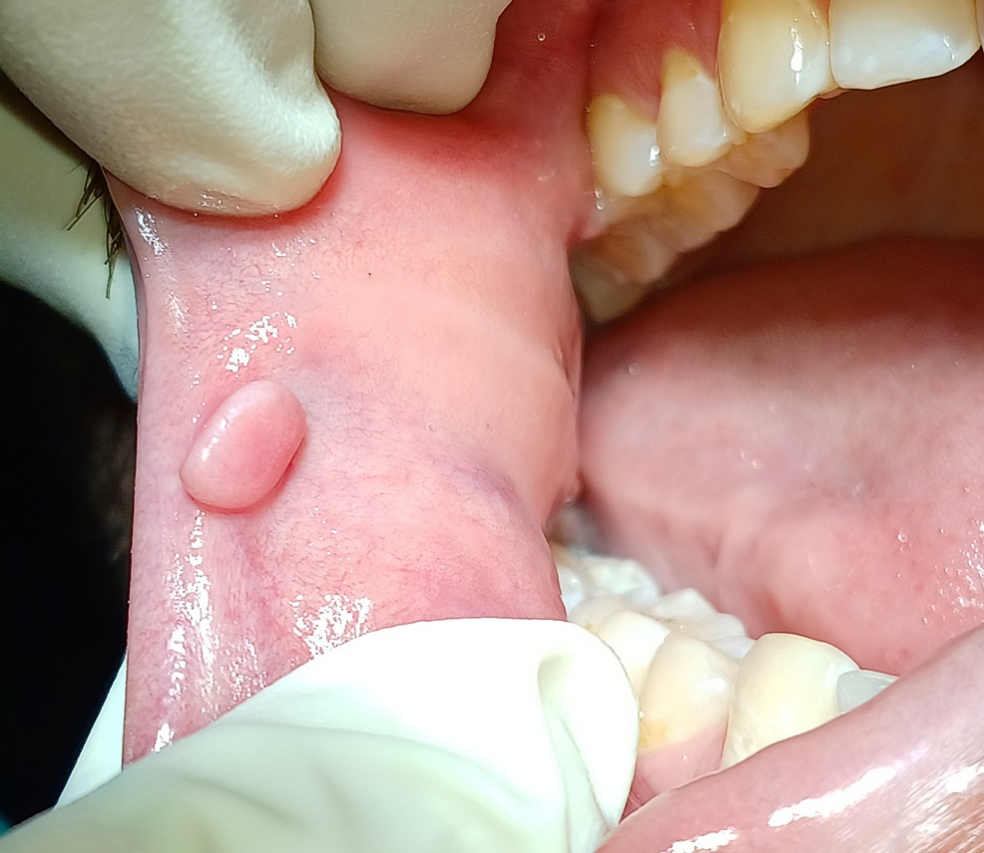
Pre-operative view of traumatic fibroma associated with buccal mucosa.

A 26-year-old male patient reported to the periodontics department with a chief complaint of soft tissue growth over the right buccal mucosa of the mouth. Considering the clinical history and examination, the diagnosis of traumatic fibroma was framed. The lesion occurred six months back. Since then, it has been present in the mouth. Initially, it was small, pedunculated, stalk-like, and firm, and gradually increased with no history of bleeding pain. The patient had a history of traumatic biting due to orthodontic treatment nine months prior, and the main aetiologic factor for the same was orthodontic bands and brackets. The overlying mucosa was normal in colour, with no evidence of ulceration. The growth was soft in consistency, sessile, and non-tender, as shown in Figure 4.

**Figure 5:**
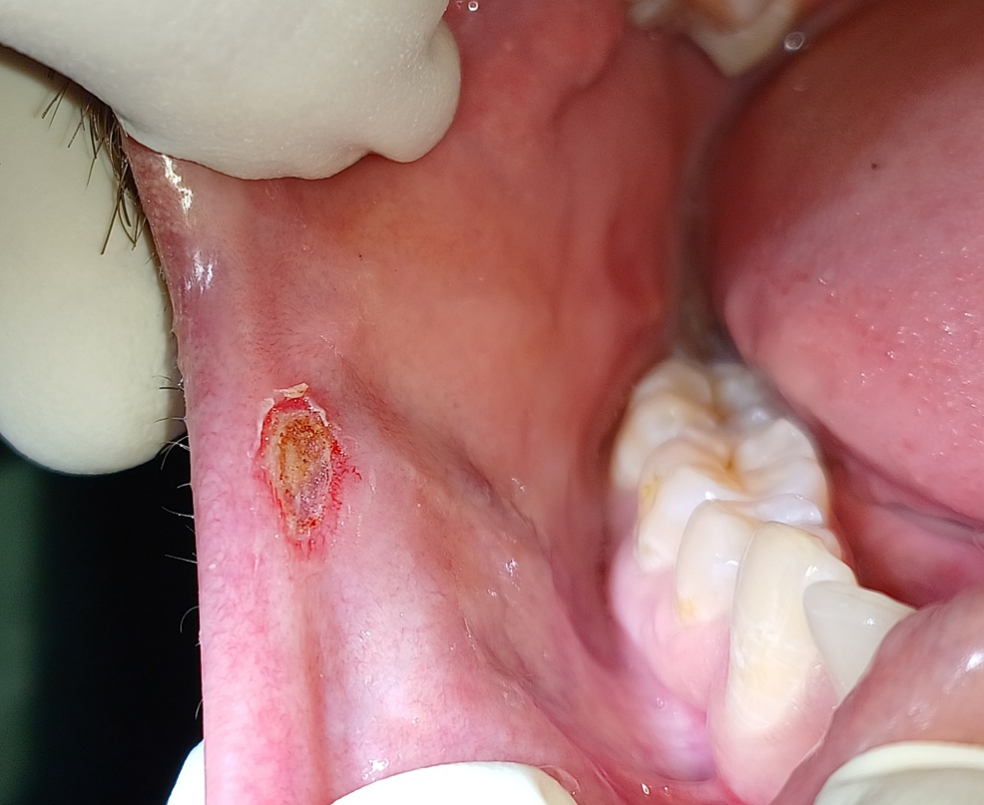
Surgical excision of traumatic fibroma was done by using a diode laser.

The lesion was 5 mm wide and 9 mm long. The patient was informed about the surgical procedure and written consent was obtained. Under local anaesthesia and by maintaining proper laser safety protocol, surgical lesion removal was done using a diode laser with a wavelength of 880 nm. Figure 5 shows no bleeding, discomfort, or pain associated with using a diode laser during surgery.

**Figure 6:**
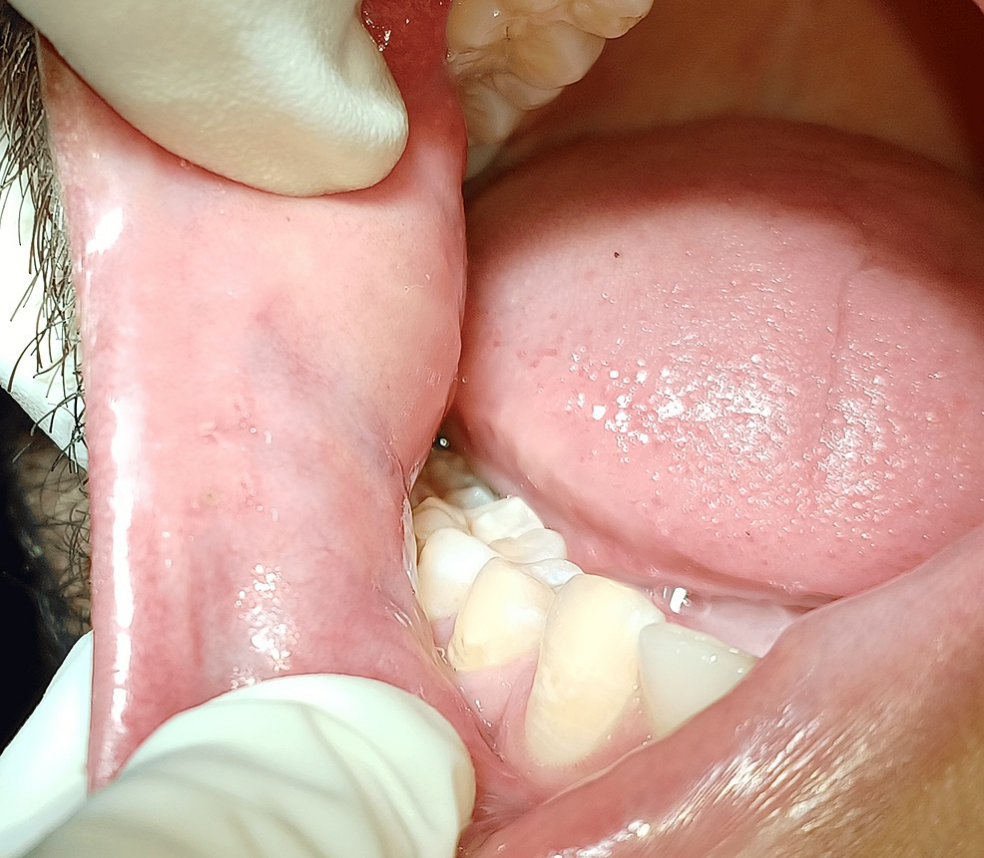
Recall examination after seven days revealed satisfactory wound healing.

The excised specimen on histopathological examination confirmed the diagnosis. The patient was recalled seven days after surgery, which revealed complete satisfactory wound healing and no scar formation, as seen in Figure 6. No recurrence signs were present on the three-month follow-up examination. Upon careful appraisal, diagnosing and treating the traumatic fibromas are needful. Traumatic fibromas in the aesthetic zones are essential to treat as they can cause traumatic occlusion and difficulty chewing and speech.

## Discussion

Various pathological processes can cause tissue enlargement in the oral cavity, leading to diagnostic difficulties. The most frequent benign lesion in the oral mucosa is traumatic fibroma, which develops from gingival connective tissue or the periodontal ligament. The growth has a smooth surface, normal-coloured mucosa, a sessile or pedunculated base, and a firm consistency. Due to decreased vascularity, the lesion appears as a round or oval, sessile, broad-based swelling that is painless and has a lighter colour than the surrounding tissue. Histopathologically, it seems to be a nodular mass composed of collagen-containing fibrous connective tissue [5]. Gingival lesions are classified as “pyogenic granuloma, peripheral giant cell granuloma, fibrous hyperplasia, fibroma, and peripheral fibroma”. The lesions consist of mature fibrous tissue with a noticeable vascular pattern. The age and inflammation level of the lesion also affect the epithelial changes. Fibroepithelial hyperplasias in inflammatory situations are covered in a consistently hyperplastic epithelium that, when ulcerated, takes the shape of an arcading rete pattern [6]. The recurrence of traumatic fibroma is significantly influenced by aetiological variables such as dental calculus, fractured restorations, occlusal trauma, sharp or uneven tooth edges, and lip-biting habits. The lesion has more middle-aged predilection, and the prevalence rate is 1.2%, with 66% female predilection. The advantage of this lesion is that no post-operative scar marks are visible after proper treatment modalities. Oral fibromas grow over weeks or months to reach their maximum size, typically 1 cm in diameter, but vary from case to case. A diagnosis may be suspected if a patient presents with typical symptoms of oral fibroma during a medical examination [7]. The lesion used to be slightly red and firm sometimes. A radiograph shows no bone involvement. Supragingival scaling in phase I therapy, followed by a week-long chlorhexidine mouthwash prescription and surgical removal or excision with a scalpel or diode laser, is being carried out [8]. There is a wide range of differential diagnoses for every lesion on the oral mucosa. Most cases of traumatic fibroma are asymptomatic. Other indications of traumatic fibroma include obstruction by the lesion during speech and mastication, in addition to progressive enlargement [9,10].

## Conclusions

This case report demonstrates that surgical excision of traumatic fibroma with a scalpel and diode laser was discovered to be a simple, efficient, and affordable method for treating traumatic fibroma and giving aesthetic and functional clearance to the patient. In contrast, the removal using a diode laser demonstrates no bleeding and less discomfort and pain during the surgery than the conventional scalpel technique. However, the scalpel method is less expensive than diode laser. The patient's clinical history and varying aetiologic factors should be considered for non-recurrence of the lesion, and the patient should be evaluated on recall and educated about the traumatic habits to stop the recurrence. This case report involves two cases of traumatic fibroma, which were reported with aesthetic concern and functionality issues during speech and mastication and regressed later on surgical intervention.
